# A retrospective cone beam computed tomography investigation of incidental findings in the maxillo-facial region

**DOI:** 10.6026/973206300212059

**Published:** 2025-07-31

**Authors:** Meenakshi Bhasin, Anshu John, Niharika Benjamin, Pooja Singh, Bhavana Yadav, Tanya Jain

**Affiliations:** 1 Department of Oral Medicine and Radiology, Hitkarini Dental College & Hospital, Jabalpur, Madhya Pradesh, India; 2 Department of Public Health Dentistry, Government Dental College and Hospital, Jamnagar, Gujarat, India

**Keywords:** Computed tomography, field of view, incidental findings, maxillofacial region, nasal and sinus pathologies

## Abstract

The current retrospective study was intended to assess the prevalence, type and location of incidental findings on CBCT images taken for various dental diagnostic desired outcomes. The scans were taken using the CS 8100 3D Select scanner with fixed parameters (60-90 kV, 2- 15 mA and exposure time 07-15 seconds). The maximum field of view (FOV) was 8 x 9cm, with a grey scale of 16384-14 bits. The archived retrospective CBCT scanned images chosen for the study were classified according to gender. Out of the 342 CBCT scans evaluated, a remarkable 300 (90.7%) indicated a total of 631 incidental results that were unrelated to the primary reason for the CBCT scan.

## Background:

Panoramic radiography is effective for presenting an overview of maxillofacial hard tissues and could uncover jaw-related pathologies. Nevertheless, panoramic radiography images have similar intrinsic limitations as conventional 2D projections, including image amplification, superimposition of anatomical or disease conditions and structural misrepresentation. To address some of the aforementioned shortcomings, cone beam computed tomography (CBCT) for the jaws evolved; in the recent past, CBCT has emerged as the primary three-dimensional diagnostic imaging technique in the maxillofacial region [1]. A conical X-ray beam circles the subject in a single or partial rotation, generating several two-dimensional (2D) projections. Utilising reconstruction methods, the obtained 2D pictures are turned into a three-dimensional (3D) database that may be examined in the axial, sagittal and coronal directions. The CBCT provides medical practitioners with a valuable tool for diagnosing different medical conditions and planning dental procedures by using features like magnification and visual enhancements (grey scale, brightness and contrast level) [2]. It has also been proposed that CBCT could detect periapical lesions at an early stage and more precisely than conventional radiography. Asymptomatic or concealed pathology may result in a delayed diagnosis, compromising the treatment approach and prognosis [3]. It has been utilized to precisely localize impacted or ectopic canines, supernumerary teeth and foreign substances. It is also used to evaluate third molar relationships, dental trauma and root fractures. Furthermore, CBCT is effective in determining anatomy, developmental abnormalities and the degree of injuries in the maxillofacial regions. Given its benefits, CBCT has drawbacks such as poor soft tissue contrast and image noise caused by scattered radiation. Because of the occurrence of false-positive lesions caused by metallic restorations, CBCT cannot be utilized reliably on the usual high-risk patient for caries. CBCT is an effective method of detecting accidental oral and maxillofacial anomalies. The large CBCT volume extending the entire maxillofacial area enables the detection of aberrant pathologies outside the targeted region of focus [4]. When analyzing CBCT scans, it is critical to analyse the full image volume rather than focusing solely on the vicinity of interest. Careful and thorough investigation enables the identification of incidental results with clinical importance. Incidental findings in CBCT scans are thoroughly documented. The actual frequency of incidental findings varies greatly between studies, contingent upon the age of the patient, population analysed and type of findings [5]. A greater knowledge of the incidental findings disclosed by CBCT scans may assist professionals in identifying clinically significant abnormalities while avoiding redundant diagnostic procedures and evaluations for lesions that are not entitled to intervention or therapies [6]. Therefore, it is of interest to retrospectively assess the prevalence, type and location of incidental findings on CBCT scans taken for various dental diagnostic needs.

## Materials and Methods:

This retrospective study was carried out in the Department of Oral Medicine and Radiology, Hitkarini Dental College and Hospital, Jabalpur and was approved by the Institutional Ethical Committee. The sample size was calculated using G Power software for a 2-group chi-square test effect. Size was calculated using the formula.

Effect Size = P2-P1

Where P1 & P2 are the proportions taken from previous literature

(P1= 14.5%, P2= 28.5%)

By setting α value at 0.05 and the β value at 0.80, the minimum estimated sample size was calculated to be 292. So, a total of 300 samples were taken for the study. CBCT scans of subjects aged between 10-79 years, selection of CBCT scans of good quality and scans indicated for evaluation of maxillary and mandibular pathology, sinus pathologies, orthodontic treatment, implant assessment and calcification were included in the study. Those records which were having missing information and were incomplete, patients having CBCT scans with orthognathic surgery because they need a larger FOV and Children below 10 years of age were excluded. The CS 8100 3D Select scanner was used with fixed parameters (60-90 kV, 2- 15mA, exposure time 07-15 seconds) and a grey scale of 16384-14 bits. The maximum field of view (FOV) was 8 x 9cm. The stored retrospective CBCT scanned images were categorised into groups based on gender. All CBCT scanned images were evaluated depending on specific parameters such as dento-alveolar region, cysts and tumours, nasal and sinus pathologies, supernumerary and impacted teeth and TMJ region and miscellaneous.

## Statistical analysis:

The recorded data was obtained from a database scan of the CS 8100 3D Select Scanner using fixed parameters. The data was collected systematically, coded in a Microsoft Excel file and statistically analysed using IBM SPSS Software version 26.0. All of the findings were tabulated and the mean and standard deviation for each parameter were computed before being statistically analysed using the Chi-Square test. The current study included both observational and comparative statistical analyses. A p-value of ≤0.05 was deemed significant.

## Results:

The average age of the study sample was 45.91±17.40 years (range: 4 to 76 years), indicating a wide variability among the participants. Of the 342 CBCT scans collected retrospectively from the database of patients, 300 CBCT scans were collected, 210 (70%) were males and 90 (30%) were female patients. These 300 (90.7%) scans disclosed 631 incidental findings that were not related to the primary reasons of the CBCT scan, while 42 scans were without incidental findings. The incidental findings were most common in the dentoalveolar region (42.49%), followed by nasal & sinus pathologies (25.91%), cysts & tumors (19.86%), supernumerary & impacted teeth (6.48%), miscellaneous (3.8%) and TMJ region (1.47%) are the least common. In our study, there was a significant relationship between FOV and incidental findings. A small FOV revealed 90.8% (n=128) incidental findings out of 141 scans, medium FOV revealed 89.6% (n=182) incidental findings out of 96 scans, whereas large FOV revealed 98.1% (n=321) incidental findings out of 63 scans. More incidental findings were found in images with a large FOV. Out of 300 scans, total incidental findings reported were 631 and the results were highly significant (p=0.000). The association between gender and dentoalveolar findings reveals that rarefying osteitis, sclerosing osteitis, enostosis, root fragments, periapical granuloma, apical periodontitis and periapical abscess were most common in males than females whereas periodontal bone loss was most prevalent in females than males, results were observed to be not significant (p=0.958) (Table 1 - see PDF). In our study, all the incidental findings, i.e., periapical cyst, residual cyst, odontoma, odontogenic cyst, dentigerous cyst and Stafne's bone cyst, were most prevalent in males as compared to females and no significant difference was observed between genders (p=0.596) (Table 2 - see PDF). In our study, mucositis, mucous retention cyst and maxillary polyp were more common in males than in females, while antrolith was found to be more prevalent in females than males; the results were significant (p=0.002) shown in Table 3 (see PDF). In supernumerary and impacted teeth, the association between gender reveals that impacted canines and impacted molars were more prevalent in males than in females and the results were not significant (p=0.918) (Table 4 - see PDF). The incidental findings observed in the TMJ region were an elongated styloid process, which was equal in both males and females, whereas coronoid hyperplasia was more common in males than females and the result was not significant (p=0.482) (Table 5 - see PDF). All the miscellaneous incidental findings, i.e., G.P beyond apex, open apex, implant impinging on nerve/implantitis and broken instrument, were more prevalent in males than in females and the results were not significant (p=0.864) as shown in Table 6 (see PDF).

## Discussion:

3D CBCT has several applications in dentistry, including maxillofacial diagnostics. It may yield useful diagnostic data beyond the area of interest. For instance, the CBCT volume may indicate inadvertent observations in maxillofacial locations, including both gnathic and extragnathic features [7]. Most CBCT equipment has limited field-of-views (FOVs) that enable investigation of sections of the oral-maxillofacial region while reducing unwanted radiation to sensitive tissues such as bone marrow, salivary glands and oral mucosa [8]. To limit the effective radiation dosage, narrow FOVs are used in CBCT image collection whenever feasible [9]. It has been demonstrated that incidental findings occur in over 90% of CBCT scans with large FOVs [10]. The significance of accidental findings from CBCT scans varies, ranging from frequent benign lesions to serious abnormalities that might have an influence on the well-being of the individual. These findings comprise normal anatomical variations, age-associated results, developmental findings and concealed pathological results [11]. Of the 342 CBCT scans evaluated, a stunning 90.7% (300) indicated a total of 631 incidental findings unrelated to the primary rationale for the CBCT scan. The vast majority of the results are benign and do not necessitate any specific intervention. This underscores the importance of carefully and thoroughly interpreting CBCT images beyond the region of interest to prevent overlooking occult pathology, which could result in serious health effects for the patient [12]. A trained and skilled CBCT scan interpretation is essential to mitigate needless surgeries (for example, in the event of a benign mimic of a significant pathology) and to diagnose pathologies promptly, hence reducing treatment time, complexities, challenges, and associated expenses [13]. Cha *et al.* [14] reported the largest frequency of incidental findings in the airway region (18.2%), followed by the TMJ region (3.4%), endodontic therapy (1.8%), and others (1.2%). Nevertheless, the dentoalveolar region (42.49%) was the most prevalent incidental finding in our sample, trailed by nasal and sinus diseases (25.91%), cysts and tumours (19.86%), supernumerary and impacted teeth (6.48%), miscellaneous (3.8%), and the TMJ region (1.47%). Price *et al.* [15] identified 881 incidental findings in 272 scans, averaging 3.2 imperfections per scan. The most common results were airway-related (35%), followed by soft tissue calcifications (20%), bone abnormalities (17.5%), temporomandibular joint issues (15.4%), endodontic conditions (11.3%), dental developmental anomalies (0.7%), and pathological conditions (0.1%). Intervention or referrals were necessary for 16.1% of the patients, while 15.6% necessitated monitoring. The remaining 68.3% of the patients demanded neither intervention nor referral. Their study findings emphasized the necessity of meticulously analyzing all CBCT data for clinically pertinent results, regardless of the region of focus. The dento-alveolar region includes rarefying osteitis, sclerosing osteitis, enostosis, apical periodontitis, periapical abscess, periapical granuloma, root fragments and periodontal bone loss. Earlier endodontic procedures or apical surgery might result in a dense fibrous scar that presents as periapical radiolucency and may resemble periapical rarefying osteitis. Rarefying osteitis is a frequent response to inflammation and infection, with microbial invasion in the root canal system being a primary cause. This inflammatory response causes bone loss and might appear as a radiolucent region on radiographs. According to Rai *et al.* [16], nasal and sinus region results were the second most common category (28.3%). The most prevalent diagnosis was mucositis/mucous retention cyst (83.4%). However, in our investigation, this region was the second most common characteristic, accounting for 25.91 percent. CBCT scans can assist in detecting mucositis, mucous retention cysts, antroliths, maxillary polyps and a deviated nasal septum. The most prevalent finding was mucositis (23.3%), subsequently followed by a mucous retention cyst (9.7%). Observations in this area are crucial when considering an implant in the posterior maxillary region. The patency of the osteomeatal complex determines postoperative problems following implant insertion and grafting procedures [17]. Mucous retention cysts occur frequently in the maxillary sinus due to their structure and the prevalence of variables that contribute to their development, including inflammation, infection and allergies. The maxillary sinus has a weak lining and numerous mucus-secreting glands, which render it prone to obstruction and eventual cyst formation [18]. Calcifications of the sinuses, or antroliths, were additionally observed. It may enhance the risk of complications after an implant or other surgical interventions performed in the sinus area. As a result, patients with nasal findings should be thoroughly evaluated for the presence or absence of sinusitis [19]. Nass Duce *et al.* [20] identified three patients with antroliths from 1957 sinus CTs analysed, which were related to sinusitis. Antroliths are calcified structures formed by mineral deposition around an endogenous or foreign nidus in the nasal cavity. Nevertheless, in our investigation, we reported two incidences (0.6%) of antroliths in two female patients, both in the maxillary sinus. According to some studies, the incidence among women is 55-60%. The exact causes of this gender predilection are unresolved, but it could be linked to hormonal variations or other unexplained physiological variations. Hormonal variations, particularly those associated with oestrogen, may contribute to the development of antroliths. Oestrogen can change tissue flexibility and immunological activity, which may influence sinus inflammation and the production of antroliths [21]. The prevalence of impacted teeth was 6.48% in the present research and 13.7% in a previous study. In our investigation, the most prevalent incidental findings in supernumerary and impacted teeth were impacted maxillary and mandibular 3rd molars (9.0%), which was consistent with the findings of Fardi *et al.* [22]. Miscellaneous findings in our study consisted of 3.8%. It involves gutta-percha beyond the apex, open apex, fractured instruments and implant impinging on nerve/implantitis. These data may give us an indication of inexplicable paresthesia or any other symptoms noticed during post-implant recall sessions [23]. In the current research, incidental findings occurring in the TMJ area were 1.47%, which is the least prevalent. Previous research found that it ranged between 2.6% and 26.5% [24, 25]. Patients presenting any accidental pathological evidence in the TMJ region could be without symptoms clinically and might not be suffering from any TMJ dysfunction [26]. The total incidence of incidental findings documented by çaglayan and Tozoglu [27] was 92.8%. The greatest proportion of incidental finding was in the airway region (51.8%), subsequent to impacted teeth (21.7%) and TMJ findings (11.1%). Nevertheless, Asaumi *et al.* [28] identified incidental abnormalities with panoramic radiography in 6.05% of children in their study. The incidental findings reported by çaglayan and Tozoglu were significantly elevated due to the inclusion of several forms of incidental maxillofacial findings, such as airway anomalies, TMJ abnormalities, impacted teeth, endodontic lesions, condensing osteitis, and various other observations. Further, Braun *et al.* [29] demonstrated that the CBCT for implant planning had a considerably higher detection rate of clinically important dental incidental findings than for other purposes (60.7% vs. 43.2%), as did CBCT with a FOV ≥ 100 mm in contrast to a FOV < 100 mm (54.7% vs. 40.0%). Comparable outcomes were observed for incidental observations in the paranasal region. In the selected subgroup evaluation, 53.7% of patients exhibited previously unreported incidental results, which altered therapeutic care in 35% of patients. These findings underscore the necessity of a thorough examination of the complete field of view of CBCT for incidental findings, which demonstrated clinical significance in almost one-third of patients. Given the substantial frequency of clinically significant incidental findings, particularly in CBCT for implant planning, a field of view measuring 100x100 mm encompassing both the upper and lower jaws is deemed advisable for this purpose. One of the limitations of the study was the absence of previous clinical, radiographic, or histologic records to ascertain whether the CBCT data had been previously diagnosed or noticed in those earlier investigations. This aspect may be the subject of future research. Lastly, there was no clinical link between the CBCT findings because this investigation only examined the scans and did not involve any interactions with patients.

## Conclusion:

Out of the 342 CBCT images evaluated, 300 (90.7%) scans indicated a total of 631 incidental findings that were not related to the primary reason for the CBCT evaluation. CBCT investigations with a large FOV may show a high rate of incidental findings in the maxillofacial area. As a result, oral radiologists and dental professionals must pay close attention to these incidental findings and thoroughly examine every detail to avert over- or underestimating the underlying conditions and deliver comprehensive health treatment to patients.
